# Association between attention performance and the different dimensions of DSM-5 depression symptoms

**DOI:** 10.3389/fpsyt.2023.1291670

**Published:** 2023-12-21

**Authors:** Ana Lucia Taboada Gjorup, Julio César Tolentino Júnior, Eelco van Duinkerken, André Casarsa Marques, Aureo do Carmo Filho, Alan Marques Joaquim, Vithória Vidotti Neves, Sergio Luis Schmidt

**Affiliations:** ^1^Post-Graduate Program in Neurology, Universidade Federal do Estado do Rio de Janeiro (UNIRIO), Rio de Janeiro, Brazil; ^2^Department of Medical Psychology, Amsterdam UMC, Vrije Universiteit, Amsterdam, Netherlands

**Keywords:** PHQ-9, depression symptoms, somatic symptoms, attention, neuropsychology, cognition

## Abstract

**Objective:**

Depressive symptoms can be assessed with self-reported questionnaires, such as the Patient Health Questionary-9 (PHQ-9). Previous studies have suggested that the PHQ-9 items can be grouped into somatic and non-somatic clusters. However, the classification of the PHQ-9 item “concentration difficulties” into somatic or non-somatic is still controversial. This controversy may be explained by difficulties experienced by subjects in accurately evaluating their attention problems. The primary objective of this study was to determine the correlation between objective attentional performance and the two clusters of depressive symptoms in hospital employees working in stressful conditions.

**Methods:**

The participants filled out the PHQ-9 to identify their depressive symptoms. Based on the PHQ-9, the somatic or non-somatic symptoms were measured without considering the question about subjective concentration difficulties. Then, a brief version of the Continuous Visual Attention Test (CVAT) was applied to assess four attentional subdomains. The CVAT is a Go/No-go task that measures number of correct responses (focused attention), number of incorrect responses (behavior-inhibition), average reaction time of correct responses (RT-alertness), and variability of reaction time (VRT-sustained attention). The entire task lasted 90 s. Correlation analyses assessed the relationships between attentional performance and the two dimensions of depressive symptoms.

**Results:**

After applying the inclusion/exclusion criteria, 359 individuals were selected. Their age ranged from 20 to 70 years (mean = 40.5, SD = 10.37), and the majority was female (67.6%). A predominance in somatic depressive symptoms was present in 231 (64%) participants, whereas 59 (16%) showed a predominance of non-somatic symptoms. Sixty-nine participants (20%) did not show any predominance. Higher somatic scores were associated with higher RTs, whereas higher non-somatic scores were related to an increase in the number of incorrect responses.

**Conclusion:**

The predominance of the somatic cluster was related to lower alertness, whereas the predominance of non-somatic cluster was associated with impulsivity/hyperactivity. This result may explain the difficulties associated with correctly classifying the item concentration difficulties. A brief attentional task can be used as an auxiliary tool to correctly identify the different dimensions of attention that are associated with different clusters of depressive symptoms.

## Introduction

Depression is a prevalent disorder (1) that cause significant functional impairment (2, 3). According to the Diagnostic and Statistical Manual of Mental Disorders, Fifth Edition (DSM-5), unipolar Major Depression Episode (MDE) requires five or more symptoms to be present for a period of at least 2 weeks (4). One of these symptoms should be either depressed mood or anhedonia (loss of interest or pleasure), with the secondary symptoms of MDE being appetite or weight changes, sleep difficulties (insomnia or hypersomnia), psychomotor agitation or retardation, fatigue or loss of energy, diminished ability to think or concentrate, feelings of worthlessness or excessive guilt, and suicidality. The diagnosis of MDE requires structured or semi-structured interviews based on these nine criteria, which are conducted by trained physicians (5, 6). In addition, several scales and questionnaires have been used to screen for MDE (7). In particular, the Patient Health Questionnaire-9 (PHQ-9) is such a commonly used self-administered instrument based on these nine criteria (7), and has been validated for the screening of MDE (7, 8).

Based on the PHQ-9, there have been several attempts to detect individuals with depression subtypes based on severity (9). However, patients with depressive disorders show a wide range of clinical manifestations, including neurovegetative and emotional-affective symptoms (2). Different symptom clusters can be essential for understanding the neurobiological substrates of major and minor depression. Therefore, it is also relevant to investigate subgroups based on different symptomatology rather than just severity. Previous studies based on the PHQ-9 have demonstrated that depressive symptoms can be classified into two dimensions: 1-“non-somatic symptoms,” a broad concept that includes core symptoms of depressed mood or anhedonia, as well as feelings of worthlessness, guilt, and suicidal thoughts and 2-“neurovegetative symptoms,” such as sleep problems. Doi et al. demonstrated that a bi-factor model is invariant between either nonclinical and clinical groups, including depressed or anxious patients (10). Moreover, Petersen et al. have shown that the best-fitting model across genders is a two-factor model with ‘somatic’ and non-somatic items (11).

Thus, the PHQ-9, depressive symptoms have been previously divided into somatic and non-somatic factors (12, 13). The items sleep difficulties, fatigue, appetite changes, and psychomotor agitation or retardation loaded on the somatic factor. However, in some studies, the item concentration difficulties loaded on the somatic dimension (2, 12, 13), and on the non-somatic dimension in others (13, 14). Therefore, the classification of the PHQ-9 item concentration difficulties remains controversial.

Part of the above-mentioned controversy may be explained by difficulties experienced by subjects in accurately evaluating their cognitive problems. It is well known that neurological and psychiatric patients sometimes report worse cognitive abilities, while their performances are within normal range on standardized tests (15). Concentration difficulties of the PHQ-9 are based on subjective judgment. Furthermore, objective assessments of attentional performance have indicated that attention is not a unitary construct, but rather it is composed of at least 4 subdomains (16, 17), possibly making reliable subjective judgment of attention capacity more challenging.

The correct classification of the attention problems in MDE is of theoretical and practical interest. Recent studies have suggested that cognitive performance can be used to monitor and predict treatment response to antidepressant drugs (18–21) and to cognitive behavioral therapy (22). In fact, more than 30% of depressed patients do not respond adequately to standard pharmacological treatment (23) and chances of remission decrease with each failed treatment attempt (24). The difficulties associated with the treatment responses may reflect that the diagnosis of MDE includes several different brain pathologies (25). This highlights the need of correct identification of suitable markers for stratifying MDE patients into clinically meaningful subgroups. Thus, we investigated how the different subdomains of objective attention performance were associated with the two dimensions of depressive symptoms.

Objective attentional impairment has been studied by examining performance on Go/No-go tasks (26). The Continuous Visual Attention Test (CVAT) is based on the Go/No-go paradigm and provides a measurement of attention subdomains (26–28). A short version of the CVAT, which takes only 90 s to complete, has been administered in several clinical scenarios (21, 29), and can be easily administered in large samples.

In certain specific situations, there are time constraints to administer long neuropsychological batteries and extensive psychiatric interviews. During the COVID-19 outbreak, hospital employees (HEs) have been compelled to keep working, even when presenting symptoms of depression and subjective concentration complaints. Previous investigations have reported that psychological distress was substantially enhanced in health care workers during the pandemic (30, 31). Therefore, there has been a need to administer self-report questionnaires and quick objective attention assessments in HEs who continued to work during the COVID-19 period. Apart from their potential use as markers, specific attentional disturbances may also constitute important treatments target in HEs with depressive symptoms (32) because impaired attentional performance negatively impacts adequate functioning and may compromise work safety (21, 33–35).

In this study, the PHQ-9 was used to evaluate depressive symptoms and to divide HEs into two groups, one with predominantly somatic and another with predominantly non-somatic depressive symptoms, without considering subjective ratings on concentration difficulties. This classification was performed with the aid of a Depression Symptoms Scale (DSS) that was calculated using the PHQ-9 items without including the item “concentration difficulties.” Objective attention performance was measured using the 90-s CVAT.

The primary objective of this study was to investigate the relationship between the DSS and CVAT performance (first objective). We assumed that the strength and direction of the relationship between CVAT performance and the DSS would indicate whether objective attentional performance loaded on the non-somatic or somatic domain. We also evaluated the magnitude of objective attention deficits by means of standardized scores based on a reference group of healthy participants assessed before the COVID-19 pandemic (second objective). Lastly, we analyzed whether there was a relationship between self-reported concentration problems and objective attentional performance (third objective).

## Materials and methods

### Participants

This study was conducted between May 12 and December 9, 2020. We included HEs between 20 and 70 years old who were working during the COVID-19 pandemic at a reference University Hospital. Exclusion criteria: previous or current SARS-CoV-2 infection, presence of neurological problems, uncontrolled clinical conditions, and the use of psychotropic medications that could interfere with objective attention performance. Educational level was classified into three levels: 1- elementary, from 1 to 8 years of formal education; 2- high school, from 9 to 12; 3- college or higher (>12 years).

### Procedures

**Figure 1:**
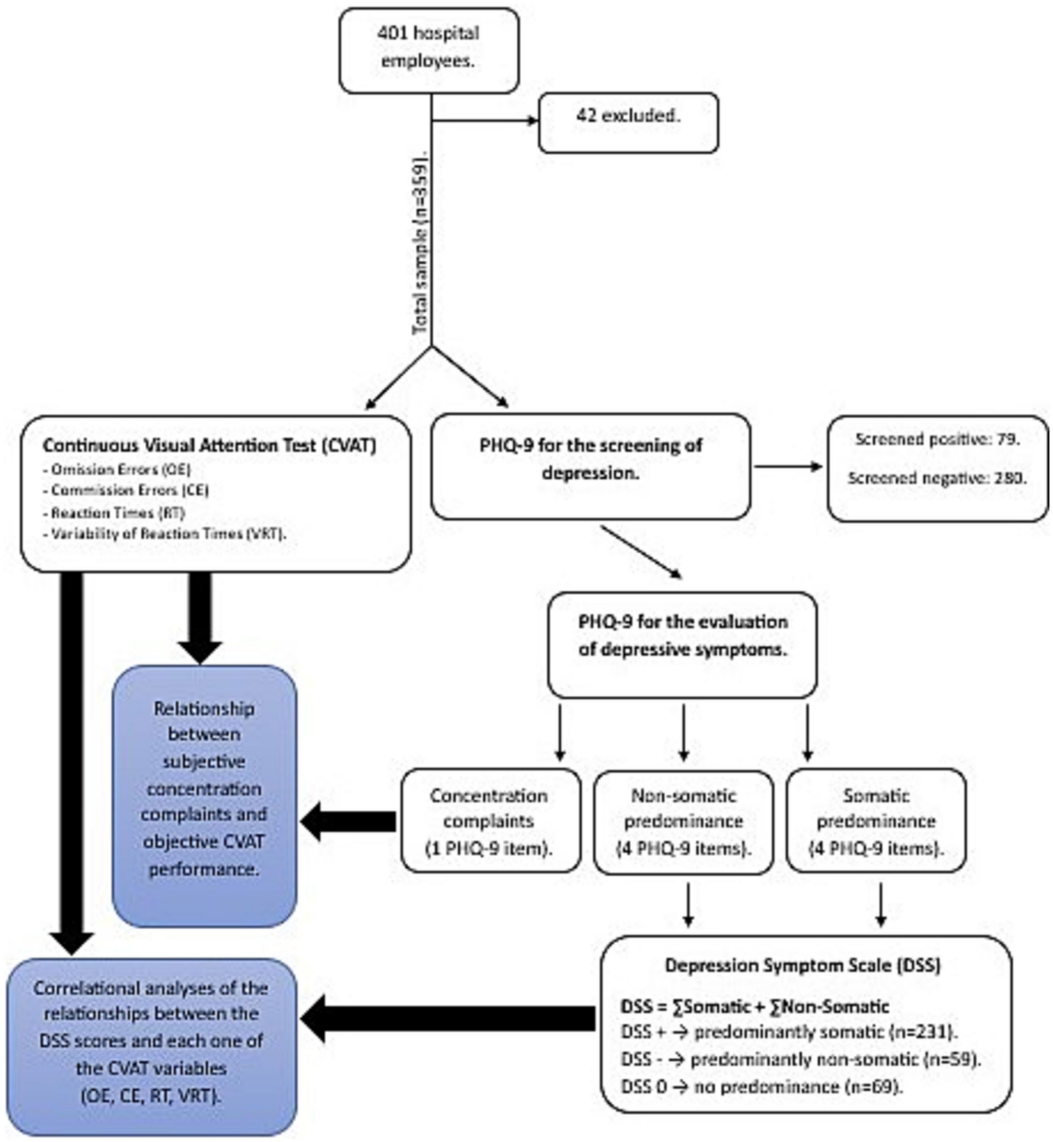
Procedures. CVAT (Continuous Attention Performance Test). PHQ-9 (Patient Health Questionaire-9). Omission Errors (OE). Commission Errors (CE). Reaction Times (RT). Variability of Reaction Times (VRT). DSS (Depression Symptoms Scale).

A flow chart of the study can be found in Figure 1. All participants filled out a self-questionnaire including demographic, clinical data, and the PHQ-9 (*n* = 401). Then, attentional performance was assessed with the short version of the CVAT. The researchers who administer the CVAT were unaware of the results of the questionnaires. After the analysis of demographic and clinical data, 42 participants were excluded since they did not meet inclusion or exclusion criteria. A total of 359 participants were included in the study of the relationship between the DSS and CVAT variables (1st objective), as well as in the determination of the magnitude of objective attention problems (2nd objective), and in the analyses of the associations between subjective and objective attention problems (3rd objective).

The PHQ-9 items related to problems with sleep, fatigue/loss of energy, appetite, and psychomotor agitation/retardation were considered as somatic depressive symptoms whereas depressed mood, anhedonia/lack of interest, worthlessness, and suicidal ideation were considered non-somatic depressive symptoms (12, 36, 37). In our analysis, the criterion involving concentration problems was excluded from both dimensions. As each item was scored 0–3, the score for each factor ranged from 0 to 12. To separate the somatic from the non-somatic factor, the scores of the non-somatic items were multiplied by - 1. Thus, the somatic scale ranged from 1 to 12, and the non-somatic ranged from −1 to −12. For each participant the final DSS score was calculated summing up the positive somatic and negative non-somatic rates and ranged from −12 to +12. A negative DSS value indicated a predominance for non-somatic symptoms, while a positive DSS score indicated somatic predominance.

Based on the DSS we divided the sample (*n* = 359) into three groups: somatic predominance (DSS > 0; *n* = 231), non-somatic predominance (DSS < 0; *n* = 59) and without predominance (DSS = 0; *n* = 69). The without predominance group included participants who got score = 0 in the two domains and those who got equal negative (non-somatic symptoms) and positive (somatic symptoms) values.

### Attention assessment: CVAT

**Figure 2:**
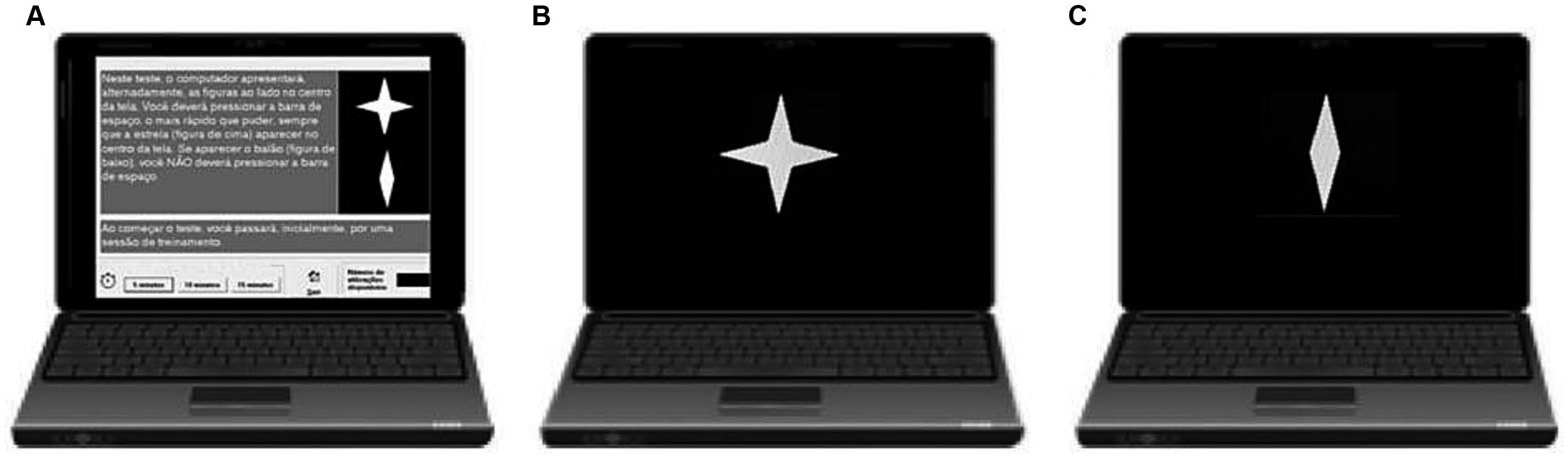
Schematic overview of the Continuous Visual Attention test (CVAT) showing the target (star) and non-target (diamond). **(A)** Instructions exhibited on the beginning of the test: “In this test, the computer alternately displays the indicated figures in the center of the screen. You must press the spacebar using your dominant hand as fast as you can whenever the star appears in the center of the screen. If the other figure appears, you should not press the space bar.” **(B)** The target remains on the screen for 250 milliseconds (ms). **(C)** The non-target also remains on the screen for 250 ms. The test consisted of 90 trials. The interstimulus time interval was 1 s. The total test took 90 s to complete. Variables: Omission Errors (OE), Commission Errors (CE), average Reaction Time of the correct responses (RT), and Intraindividual Variability of Reaction Time (VRT). The CVAT is open for research and for clinical use for licensed psychologists, upon request to Prof. Sergio L. Schmidt (corresponding author). There are versions in English, Spanish, and Portuguese. CVAT: Continuous Visual Attention Test. [Adapted from (28)].

To evaluate attention performance, a 90-s computerized Go/No-Go test was used. A practice session was presented before testing commenced, in which no errors could be made. The target stimulus consisted of a star presented in the middle of the screen, whereas the non-target stimulus was a diamond. This test consisted of one block of 90 trials, each trial being presented for 250 milliseconds (ms), with an interstimulus interval of 750 milliseconds and a stimulus onset asynchrony of 1 s. Of the 90 trials, 72 (80%) were targets (stars), and 18 (20%) were non-targets (diamonds). The CVAT was graphically represented in Figure 2. The test assessed four variables: omission errors, commission errors, average reaction time of correct responses (RT), and variability of correct reaction times (VRT). VRT was estimated by a per-person measure of the standard deviation (SD) of individual RTs for the correctly signaled targets. The participants had to reach more than 50% of the total correct hits (minimum number of correct RT measurements per participant = 37). Those who did not reach this criterion were discarded from the study.

### Depression screening

The PHQ-9 was used to screen for MDE (7, 8). PHQ-9 quantifies the frequency, over the past 2 weeks of each of the nine DSM-V criteria for depression on a 4-point Likert scale as follows: not at all (0), several days (1), more than half the days (2), or nearly every day (3). Responses are summed to create a score between 0 and 27 points. A unipolar MDE was considered present if five or more of the nine depressive symptom criteria were present, at least “more than half the days” in the past 2 weeks, and one of these symptoms was depressed mood or anhedonia. One of the symptom criteria (suicidality) counts if present, regardless of duration (7, 8).

### Reference group and Z-scores for the CVAT variables

The reference group consisted of a subsample of subjects who were taking a mandatory medical and psychological exam for a certificate of fitness to drive before the pandemic (29). All subjects taking the mandatory exam were invited to participate in a large national study for CVAT performance. Those who agreed to participate performed the CVAT on the same day and at the same place as the mandatory health exam. We only included approved subjects (*n* = 211), with a normal neurological exam, absence of visual and hearing impairments, no psychiatric complaints, and a mini-mental status examination within the normal range.

We calculated the means and standard deviations of the reference group according to the following age intervals in years: [20–30], [30–40], [40–50], [50–60], [60–70]. For each attention subdomain, the standardized scores (Z-Scores) were calculated as follows:

- Zi = (Xi-Mean)/ SD, where:
- Zi = Z-score of the i-th participant,
- Xi = Raw CVAT score of the i-th participant.
- Mean and SD = values of the reference group according to the age of the i-th participant.

Thus, the Z-score of each participant shows how many standard deviations the participant’s performance was above or below the mean of the healthy reference group of the same age.

### Clinically relevant objective attention impairment and subjective concentration problems

The presence of an objective clinically relevant attention problem was defined by the presence of at least one CVAT variable with a Z-score > 1.64. If Zi > 1.64 for a particular CVAT variable, the i-th participant was considered impaired in that CVAT variable (complementary cumulative probability and percentile for a 1.64 Z-Score = 5%).

The presence of subjective attention complaints was defined as a score of 1 or higher for the PHQ-9 concentration question.

### Summary of the variables used in this study

Here, we have the following variables:

- four binomial variables: sex (male or female); depression screening (positive or negative); dichotomized subjective concentration problem (yes or no); dichotomized objective attention problems (yes if at least one Z-score > 1.64 or no if all Z-scores <1.64).
- ten continuous/interval variables (age, years of formal education, the four CVAT variables, and the respective four Z-scores for the CVAT variables).
- two ordinal variables (DSS and subjective concentration difficulties based on the PHQ-9 rating scale).

### Statistical analyses

Demographic variables were analyzed using independent sample *t*-tests for normally distributed continuous variables or chi-square tests for categorical variables.

Correlation coefficients were used to measure the strength and the direction of the relationships between two variables. Pearson product–moment correlation coefficients (r) were applied for measuring the relationship between two continuous variables. When one variable was continuous or ordinal and the other was nominal with just two categories, we performed the point-biserial correlation coefficient. When one variable was continuous and the other ordinal, the use of Kendall’s coefficient of rank correlation tau-sub-b (τb), is indicated. For large sample sizes, however, the performance of the Spearman rank correlation coefficient (ρ) is comparable to that of Kendall’s τb. Therefore, we only included the results based on the Spearman rank correlations.

We first determined if there was any significant relationship between demographic variables (age, education, sex) and the DSS score. In case of an absence of any significant relationship, we proceeded to verify the association between each CVAT variable (omission errors, commission errors, RT, and VRT) and the DSS. We repeated the same analyses splitting the whole sample into two subgroups based on the PHQ-9 cut-offs: one subgroup that consisted of HEs screened positively for MDE and a second subgroup that only included participants negatively screened for MDE. Correlational studies were separately performed for each subgroup. All the analyses using the CVAT variables were performed using the Z -Scores, to avoid any interference of age. We verified the correlation between subjective concentration problems based on PHQ-9 ratings of this particular item (0, 1, 2, or 3) and objective measurements of the attention subdomains based on the Z-Scores.

For each participant we also verified the association between subjective concentration complain (yes or no) and objective clinically relevant attention deficit (yes or no), as defined in the section 2.7. Chi-squares were used to measure the association between these two dichotomous variables. Agreement was estimated by kappa statistics.

SPSS Statistics for Windows, version 25.0 (IBM Corp, 2017), was used for statistical analyses. Significant level was settled at 5% (two-tailed). When necessary, correction for multiple comparisons were performed with the Bonferroni method. We checked the assumptions of the regression analysis.

### Ethics statement

Written informed consent was obtained from each participant. This study was approved by the local Ethics Committee (CAAE: 30547720.3.0000.0008), which was conducted in accordance with the Declaration of Helsinki as revised in 1989.

### Transparency and openness

We reported how we determined all data exclusions, and all measures in the present study. Data were analyzed using SPSS Statistics for Windows, version 25.0 (IBM Corp, 2017). This study’s design and its analysis were not pre-registered.

We confirm that there is sufficient information for an independent researcher to reproduce all the reported results. We also confirm that there is sufficient information for an independent researcher to reproduce all of the reported methodology. All data are available upon request to the corresponding author.

## Results

### Demographics and clinical characteristics

The total sample included 359 eligible participants (Table 1). Their age ranged from 20 to 70 years (mean = 40.5, SD = 10.37), and the majority was female (*n* = 243; 67.6%). Educational level was distributed as follows: 1- elementary (0.65%), 2- high school (11.5%), 3- college or higher (87.2%). Seventy-nine participants (22%) were screened as MDE. The mean DSS in the total sample was 1.51 (SD = 2.42, minimum −7, maximum +12). In the subgroup of participants positively screened for MDE, the DSS ranged from −7 to +12 (mean = 1.67, SD = 3.0), whereas in the non-MDE subgroup the DSS ranged from −5 to +8 (mean = 1.47, SD = 2.2). There were no statistically significant demographic differences among the total sample and the two MDE subgroups. Suicidality was found in 22 participants and 50% of them showed non-somatic DSS predominance (*n* = 11), 36% (*n* = 8) somatic predominance, and 14% (*n* = 3) no-predominance (minimum −7, maximum +5).

**Table 1:** Socio-demographic characteristics of the samples.

	Total (*N* = 359)	Screened positive for depression (*N* = 79)	Screened negative for depression (*N* = 280)
Sex (m/f; % male)	116/243 (32.3)	15/63 (18.9)	101/179 (38.1)
Age (years, mean ± SD)	40.5 ± 10.3	39.8 ± 10.7	40.8 ± 10.5
Educational level	1 (0.65%)	1 (2.5%)	1 (0.3%)
	2 (11.5%)	2 (5.0%)	2 (11.0%)
	3 (87.2%)	3 (88.6%)	3 (87.1%)

Educational level was classified as follows: 1- elementary, from 1 to 8 years of formal education; 2- high school, from 9 to 12; 3- college or higher >12 years.

### Associations between each demographic variable and the DSS

There was no statistically significant association between demographic variables and the DSS, either in the total sample or in MDE and non-MDE subgroups (Supplementary material, Table 2).

**Table 2:** Correlations between depressive symptoms scale and demographic variables.

Demographic variables	Whole sample	Screened positive for depression	Screened negative for depression
Age	*ρ* = 0.029; *p* = 0.61	*ρ* = 0.10; *p* = 0.39	*ρ* = 0.02; *p* = 0.79
Sex	r_pb_ = −0.04; *p* = 0.53	r_pb_ = 0.13; *p* = 0.27	r_pb_ = −0.005; *p* = 0.47

*ρ*: Spearman correlation coefficient, r_pb_: point-biserial correlation coefficient.

### Relationship between the DSS and attention performance (1st objective)

#### Total (n = 359)

**Figure 3:**
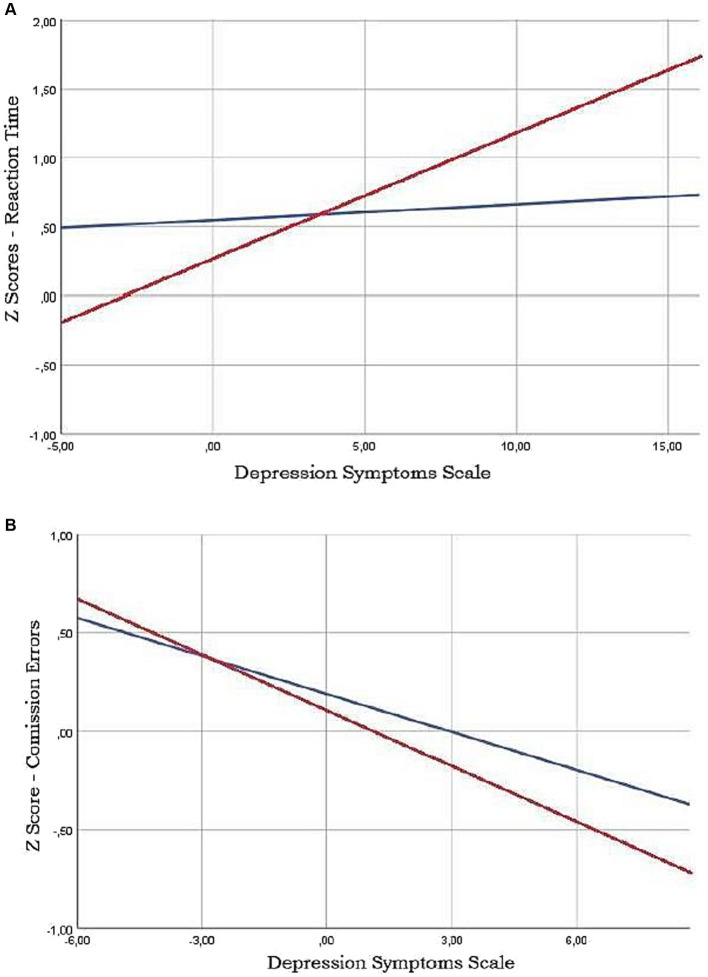
Least squares regression lines showing the relashionships between Continuous Vision Attention Test (CVAT) parameters and Depression Symptoms Scale (DSS). Note that Reaction time (RT) is positively related to DSS in patients screened as depressive by the PHQ-9 scale (red line) **(A)**. Conversely, there is a negative relationship between Commission Erros (CE) and DSS, specially in the depressive group **(B)**.

In the total sample there was a significant positive association between the DSS and RT Z-scores (*ρ* = 0.13, *p* = 0.02), and a significant negative association between Z-scores for commission errors and the DSS (*ρ* = −0.13, *p* = 0.02). In contrast, the correlations between the DSS and the other variables of the CVAT did not reach statistical significance (Figure 3).

#### Subgroups (participants classified according to MDE screening status)

Considering the total sample, there was a significant positive association between the DSS and Z-scores for RT in the MDE subgroup (*ρ* = 0.31, *p* = 0.007). For Z-scores of commission errors, a tendency for significance was found for a negative association both in the MDE (*ρ* = −0.19, *p* = 0.10) and in the non-MDE subgroups (*ρ* = −0.10, *p* = 0.09). No other correlations reached significance.

### Prevalence of relevant objective attention impairment (2nd objective)

Regarding objective attention problems, 32% of the total sample of participants (114/359) had an objective attentional impairment (Z > 1.64 in at least on attention subdomain), with the following subdomain distribution: impaired VRT: *n* = 53; impaired omission errors: *n* = 52; impaired RT: *n* = 44; and impaired commission errors: *n* = 25.

### Associations and agreement between objective attention performance and subjective concentration ratings (3rd objective)

Among the total sample (*n* = 359), there were 212 participants (59%) who reported PHQ-9 subjective concentration problems (rated 1, 2 or 3 for the concentration question of the PHQ-9).

All correlation coefficients for the relationships between subjective concentration difficulties (0, 1, 2, or 3) and Z-Scores of the CVAT variables were lower than 0.10, and not statistically significant. Accordingly, there were no statistically significant associations between Z-scores of the CVAT variables and PHQ-9 self-reported concentration problems in the total group or in the two subgroups.

As mentioned, 212 participants (59%) reported PHQ-9 subjective concentration problems and 114 participants (32%) were considered objectively impaired. Agreement between subjective concentration difficulties and objective attentional performance in the CVAT (Yes-Yes and No-No) was found in only 48% of the sample (*n* = 173). Accordingly, the kappa score did not reach significance (kappa = 0.028, *p* = 0.54). In addition, the association between dichotomized subjective concentration complains and objective dichotomized attention problems did not reach significance (chi-squared = 0.25, df = 1, *p* = 0.61).

## Discussion

We found a significant positive relationship between the DSS and RT of the CVAT. Conversely, a negative association was found between the number commission errors and the DSS. One-hundred and fourteen participants (32%) showed significant objective attention problems. There was no agreement between subjective concentration complains and objective attention deficits.

### Main objective: associations of objective attention performance with somatic and non-somatic dimensions of depression symptoms

The interpretation of the results of the first objective depends on the psychological meaning of each CVAT variable. Attention can be defined as a focused activation of the central nervous system, that enhances selective processing of information in a goal-consistent manner (38–40). Clinical data in Go/No-go tasks have supported that the attention domain consists of four relatively independent subdomains (17, 41–43): intrinsic alertness (a), sustained attention (b), focused attention (c), and behavioral inhibition (d). Previous investigations have demonstrated that impaired performance for the CVAT is related to these four subdomains as follows: (a) a drop in adequate brain activation causing a slowing of the RTs (intrinsic-alertness subdomain); (b) occasional lapses in attention, affecting the stability of RTs as the test progresses which causes an increase in VRT (sustained-attention subdomain); (c) failure of focused attention, severe enough to result in a high number of omission errors (focused-attention subdomain); and (d) inability to control inadequate responses (response-inhibition subdomain) resulting in a high number of false hits (commission errors). Therefore, the associations of the different attention subdomains with depressive symptoms depended on the nature of predominance of these symptoms (non-somatic or somatic) as discussed below.

### Positive relationship between DSS and RT

As mentioned, the RT variable of the CVAT is considered to reflect the intrinsic alertness attention subdomain (17). Therefore, the positive relationship between RT and DSS indicates that participants with higher somatic symptoms presented worsening alertness subdomain.

Although we did not perform neuroimaging exams, this finding suggests that participants with somatic symptoms presented a deficit in the alertness subdomain suggests that somatic depressive symptoms may reflect deficits in brain circuits associated with the RT variable of the CVAT. Previous functional neuroimaging studies have reported that the metabolism in the anterior cingulate cortex and the brain stem are negatively correlated with RT in Go/No-go paradigms (44, 45). Of note, these regions were also found to constitute what has been coined the central autonomic network (46). As dysfunctions of this network may also disrupt autonomic functions and cause somatic dysfunctions, this could explain the positive relationship between somatic depressive symptoms and RT. One implication of this finding is that the RT deficits in subjects with depressive symptoms may be associated with abnormal autonomic functioning.

### Negative relationship between commission errors and the DSS

The negative relationship between commission errors and the DSS indicates that behavioral inhibition might be related to non-somatic symptoms. Previous studies have suggested that impulsivity is associated with suicidality (47–50). The fact that we did not find a significant increase in the negative relationship using the depressive subsample might indicate that some moderately depressed patients suffer from anticipatory anxiety, leading to an increase in their commission errors, due to anxiety rather than due to impulsivity associated with suicidality. Despite the possible effect of anticipatory anxiety, our data suggest that subjects with high commission errors should be screened for suicidality.

### Possible implications of mapping objective attentional performance as a useful stratification tool in MDE

The finding of a negative association with the DSS and one attention subdomain (commission errors) and a positive association for another subdomain (RT) may explain why the item concentration difficulties of PHQ-9 loaded on the somatic dimension in some studies and on the non-somatic dimension in others. The results in these previous studies might reflect differences in the samples of depressed subjects, resulting in different cognitive profiles.

Cognitive dysfunction and attention impairments are well-documented in depressed patients (21, 51). Previous studies have stratified cognitive performance of depressive patients in cold and hot cognitive domains (20, 52). Cold cognition was defined as mental processes that occur independently of emotional states, such as processing speed. Hot cognition included non-somatic, emotional and motivational mental states, such as false alarms and behavioral inhibition. Based on our results we speculate that cold cognition might be relate to the somatic dimension and could be measured with RT. Conversely, hot cognition might be related to behavior inhibition and could be measured by the number of false alarms (commission errors).

A recent study showed that moderate depression was related to somatic factors while severe depression was associated with non-somatic symptoms (53). In moderated depression, previous studies have indicated that heart rate variability (46) and QT dispersion (54, 55) are most affected in moderately depressed patients. These studies determined the severity of depression based on the Hamilton Depression Scale (HAM). Although we did not use the HAM scale in the current study, most depressed participants in our study might likely present mild or moderate depression. This is supported by the fact that they kept working even with depression symptoms, which is more plausibly to happen in moderate than severe depression. Taking together, it is possible that HEs with moderate depression and slower responses might also present higher cardiovascular risk. Thus, we suggest that the pharmacological treatment of depressive subjects with alertness deficits (high RT) should not include drugs that can alter the QT interval.

**Figure 4:**
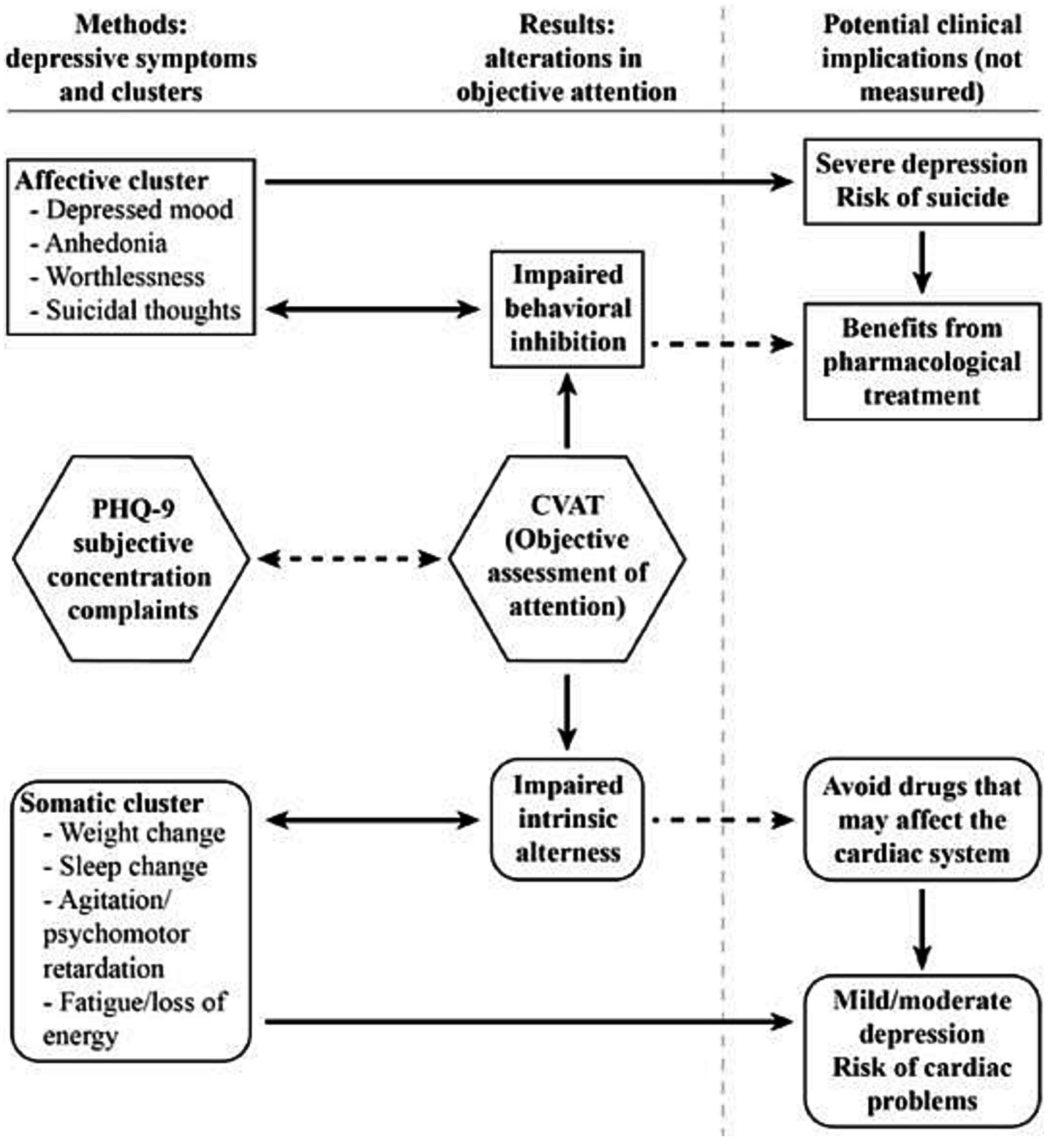
Summary of the results and possible clinical implications.

Recently Lau and collaborators (22) have demonstrated that cognitive behavioral therapy is effective in treating patients with mild to moderate depression. Conversely, subjects with an increase in commission errors might benefit from pharmacological treatment, sometimes including even antipsychotics (56). The differences in loading between these two attention subdomains, may reflect distinct attention subdomains profiles related to specific neurochemical phenotypes. Future studies should be conducted to investigate whether patients with a certain subdomain deficit may respond better to specific antidepressant drugs or non-pharmacological treatments. It would also be of interest to examine whether an early treatment response may be improved through antidepressant treatment combined with cognition-enhancing drugs (57). The results of the first objective of this study and their potential clinical implications are summarized in Figure 4.

It should be mentioned that despite the highly significant statistical levels achieved by the associations with the DSS and two CVAT variables (RT and commission errors), the correlation coefficients were modest. These results may reflect that our sample did not include a large number of clinically depressed subjects.

### Prevalence and clinical significance of objective attention deficits (2nd objective)

The prevalence of objective attentional deficits among HEs who had to keep working during the COVID-19 pandemic (32%) raised concerns about their actual cognitive abilities. Whether the objective attention deficits described here are related to a greater risk of work accidents needs to be determined in future studies. In this regard, previous investigations have reported that attentional performance measured in reaction-time tasks is correlated with safe driving indexes (33).

Additionally, our study stressed the importance of the investigation of subgroups based on different symptomatology rather than severity to identify clinically relevant subtypes. In this regard, a recent investigation examined inflammation in the context of the depressive dimensions of the PHQ-9 inventory (58). These authors found an association between inflammation and the somatic symptoms of depression, independent of cognitive symptoms (PHQ-9 item, concentration difficulties). However, the concentration item of the PHQ-9 does not consider the different dimensions of attention. The present study suggests that RT can be added to the somatic cluster. Future research should be conducted to analyze the relationship between RT and inflammatory markers in the blood, such as interleukin (IL)-1β, IL-6, tumor necrosis factor (TNF)-α, and C-reactive protein (CRP).

### Agreement between subject ratings of concentration problems and objective attention deficits (3rd objective)

The high number of subjects with subjective concentration complaints without a correlation with objective attention deficits highlighted the need for quick assessments of objective attention deficits in this population. Furthermore, our finding of a high percentage of participants positively screened for depression (22%) may indicate that the use of self-report questionnaires can overestimate depression prevalence, as suggested by Thombs and collaborators (59). In this regard, the subjective misinterpretation of concentration problems of the PHQ-9 questionnaire may contribute, at least partially, to the overestimating of depression prevalence.

## Limitations

One limitation of this study was the use of only a self-report questionnaire to screen for depression. Further studies should be conducted using structured interviews in HEs positively screened for depression. Another limitation is that we did not assess severity of depression. Futures studies should include, for instance, the use of the Hamilton Depression Scale. Although the focus here was on depressive symptoms rather than depression severity, it would be of clinical interest to verify the associations between each cluster of depressive symptoms with depression severity.

## Strengths

A strength of this study was the use of a large sample of HEs, all of them without previous or present SARS-CoV-2 infection. Another strength of this study is that a reaction-time task (CVAT) was able to identify attention deficits in HEs with depression symptoms. The CVAT is quick (90 s), requires little training, involves minimal linguistic capabilities, and provides cost-efficient diagnostics (open to licensed psychologists).

## Conclusion

Deficits in intrinsic alertness (high RTs) were related to somatic depressive symptoms, whereas deficits in behavioral inhibition (high number of commission errors) were associated with non-somatic depressive symptoms (first objective). There was a high prevalence of HEs with objective attention problems (second objective). Finally, there was no agreement between objective attentional performance and self-report concentration difficulties (third objective).

## Data availability statement

The raw data supporting the conclusions of this article will be made available by the authors, without undue reservation.

## Ethics statement

The studies involving humans were approved by Hospital Universitário Gaffrée e Guinle. The studies were conducted in accordance with the local legislation and institutional requirements. The participants provided their written informed consent to participate in this study.

## Author contributions

AT: Conceptualization, Data curation, Formal analysis, Investigation, Methodology, Supervision, Writing – original draft, Writing – review & editing. JT: Conceptualization, Data curation, Investigation, Methodology, Project administration, Supervision, Writing – original draft, Writing – review & editing. ED: Data curation, Formal analysis, Writing – review & editing. AM: Writing – review & editing. AC: Writing – review & editing. AJ: Writing – review & editing. VN: Writing – review & editing. SS: Conceptualization, Data curation, Formal analysis, Investigation, Methodology, Supervision, Writing – original draft, Writing – review & editing.
