# Supplementary material for: Association between attention performance and the different dimensions of DSM-5 depression symptoms
Source: Front Psychiatry. 2023 Dec 21;14:1291670. doi: 10.3389/fpsyt.2023.1291670 (PMC10765948; doi:10.3389/fpsyt.2023.1291670)
Supplement: Supplementary file 1 [file Data_Sheet_1.docx]

**SUPPLEMENTARY MATERIAL (SM)**

**This SM is divided into two sections:**

1. **Findings using a restricted sample (n=311)**
2. **Correlations between Depressive Symptoms Scale and demographic variables in the total sample (n=359)**
3. **Restricted sample**

We also performed a separate analysis excluding from the total selected sample (n=359) 48 participants who showed somatic (e.g., somatic=+7) and non-somatic symptoms (e.g., non-somatic=-7) without any predominance (DSS=0). In this restricted sample, we only included participants with DSS =0 if they self-reported total absence of both somatic (somatic=0) and non-somatic (non-somatic=0) depressive symptoms. This restricted sample included 311 participants (Fig. 1, SM).

Figure 1 SM: Procedures. CVAT (Continuous Attention Performance Test). PHQ-9 (Patient Health Questionaire-9). Omission Errors (OE). Commission Errors (CE). Reaction Times (RT). Variability of Reaction Times (VRT). DSS (Depression Symptoms Scale).

Their age ranged from 20 to 70 years (mean=41.0, SD=10.57**)**, and the majority was female (n=205; 65.7%). Educational level was distributed as described in the total sample. Sixty-eight participants (32.2%) were classified in the MDE group according to the PHQ-9 screening. Mean DSS in this restricted sample was 1.7 (SD=2.50). In the subgroup positively screened for MDE, DSS ranged from -7 to +12 **(**mean=1.94, SD=3.2), whereas in the non-MDE subgroup DSS ranged from -5 to +8 (mean=1.67, SD=2.3). There were no statistically significant demographic differences between the two MDE subgroups. We did not find any statistically significant association between demographic variables and the DSS in the restricted sample.

There was a significant positive association between the DSS and RT Z-scores (ρ=0.16, p=0.004), and a significant negative association between Z-scores for commission errors and the DSS (ρ=-0.16, p=0.006). Similarly, to the total sample data, the correlations between the DSS and the other variables of the CVAT did not reach statistical significance.

*Subgroups (participants classified according to MDE screening status)*

In the restricted sample, there was a significant positive association between the DSS and Z-scores for RT in the MDE group (ρ=0.28, p=0.02). For Z-scores of commission errors, a tendency for significance was found for a negative association both in the MDE (ρ=-0.22, p=0.07) and in the non-MDE subgroups (ρ=-0.13, p=0.05). No other correlations reached significance.

*Conclusions based on the restricted sample*

The data on the associations between the DSS and the four different CVAT variables did not depend on the criterion used to include participants with a DSS equal to zero. Moreover, in both samples (total and restricted), the associations were stronger in the subsample positively screened for depression. Taking together, these finding indicate that the two dimensions of depressive symptoms were associated with different attention subdomains, and that the specific associations were consistently stronger in subjects with more depression symptoms.

1. **Correlations between Depressive Symptoms Scale and demographic variables in the whole sample.**

There was no correlation between the Depressive Symptoms Scale with age and sex. We did not include years of formal education because most of the participants had the same educational level (Table1).

Table 1:

| Demographic variables | Whole sample | Screened positive  for depression | Screened negative  for depression |
| --- | --- | --- | --- |
| Age | ρ=0.029; p=0.61 | ρ=0.10; p=0.39 | ρ=0.02; p=0.79 |
| Sex | r_pb_=-0.04; p=0.53 | r_pb_=0.13; p=0.27 | r_pb_=-0.005; p=0.47 |

ρ: Spearman correlation coefficient, r_pb:_ point-biserial correlation coefficient. Note that.
